# The impact of distress disclosure and anxiety on the association between social support and quality of life among Chinese women with systemic lupus erythematosus

**DOI:** 10.3389/fpsyt.2022.893235

**Published:** 2022-08-04

**Authors:** Rui-Chen Gao, Li Wu, Pei-Li Shi, Ni Sang, Min Hao, Guo-Cui Wu

**Affiliations:** ^1^School of Nursing, Anhui Medical University, Hefei, China; ^2^Department of Rheumatology, The First Affiliated Hospital of Anhui Medical University, Hefei, China

**Keywords:** disclosure, anxiety, social support, quality of life, systemic lupus erythematosus

## Abstract

The evidence on the relationship between social support and quality of life in female systemic lupus erythematosus (SLE) patients is complex. The purpose of this study was to explore the impacts of distress disclosure and anxiety on the association between social support and quality of life among Chinese women with SLE. A cross-sectional study was conducted, and 237 samples were obtained. Measures included demographic characteristics, Lupus Quality of Life (LupusQoL), social support rate scale (SSRS), distress disclosure index (DDI), and self-rating anxiety scale (SAS). Descriptive statistics, correlation analysis, and moderated mediating effect analysis were carried out. The LupusQoL was negatively correlated with age, systemic lupus erythematosus disease activity index (SLEDAI), DDI, and SAS. SSRS had a positive predictive effect on the LupusQoL, while SLEDAI and DDI had the opposite effect. SAS had a negative predictive effect on the LupusQoL. There were interactive effects of SAS and DDI on LupusQoL. In the moderated mediation model, SAS played moderating effect in the role of DDI on LupusQoL; the DDI of female patients with SLE played a partial mediator role, the mediation effect was 0.19, and the mediation effect ratio was 33.3%. In conclusion, to pay attention to the QOL, we should consider the mediator role of distress disclosure and the moderating role of anxiety.

## Introduction

Systemic lupus erythematosus (SLE) is a typical autoimmune disease, with systemic multi-system and multi-organ involvement, recurrence, and remission alternately. So far, the pathogenesis has not been fully understood, but it may be related to the environment, hormones, and nutrition (1). Its prevalence is estimated to be 6.5–178.0 per 100,000 people, with an annual incidence ranging from 0.3 to 23.7 per 100,000 people (2). The prevalence varies by population; African Americans and Asians are more likely to have SLE (3). The prevalence rate in China is about 30–70 per 100,000 people, and the ratio of prevalence between men and women, especially in women of childbearing age (4) is about 1:10–12 (5). SLE is still uncurable. SLE patients experience significant negative emotional effects during treatment as a result of adverse factors such as recurrent episodes, unsatisfactory treatment effect, and heavy economic burden, which seriously affect the physical and mental health of middle-aged and young women. Although there has been continuous improvement in the level of diagnosis and treatment and the survival rate of patients with SLE has been greatly improved (6), the quality of life is still very low, which is lower than that of other chronic diseases (4). Quality of life has become an important index to evaluate the long-term prognosis of patients with SLE (7).

The findings of this study suggest that socio-demographic factors, such as older age, overweight, occupation, income, payment, marriage, and educational level, are major predictors of poor QoL in patients with SLE. SLE clinical manifestations, such as disease activity, disease duration, and organ damage, are also important factors in QoL (8–10). Studies have shown that psychological factors also affect the quality of life in patients with SLE (11).

Social support was conducive to actively facing the disease, effectively reducing psychological distress, and improving the quality of life (12), and has been found to alleviate the negative impacts of chronic diseases on QOL (13). There is substantial evidence that SLE affects Asian and indigenous populations as well as people of African descent more frequently; it has a more severe course, causes more organ damage, and has a higher mortality rate than whites. Poor social support is common in the non-white population and has a negative impact on the course and outcome of SLE (14). The quality of life in the United States of America, Canada, Asia, and Europe differed by gender. Women with SLE have more social support, but their functional tendencies in physical health and pain-vitality are poor (15). Furthermore, the level of social support was closely related to the quality of life in Chinese patients with SLE (11). However, the role of social support on the quality of life in patients with SLE is unclear.

As a predictor of mental health, the distress disclosure index (DDI) is the choice of emotional concealment and emotional exposure. It is the way an individual deals with emotions; patients may deal with humiliation by concealing their illness (16). It is a fundamental interpersonal process that is influenced by a variety of factors, including the targets of disclosure, the depth or type of information to be conveyed, and the breadth or amount of information (17). According to the findings of functional magnetic resonance imaging, self-disclosure can activate both reward-related and social-cognition-related brain regions, particularly those related to psychological internalization and point of view selection, implying that self-disclosure is a rewarding behavior with intrinsic value to the subject (18). In the field of chronic diseases, several studies have shown that improving quality of life can be achieved by improving self-disclosure (19, 20). A study by Corsetti et al. showed that Tandem-Psychotherapy can improve the level of distress disclosure of autoimmune patients with high levels of pain and related psychiatric comorbidities, reduces psychological distress, and improves the quality of life (21). Therefore, we speculate that the level of pain manifestation may affect the quality of life. In patients with SLE, fatigue, memory or concentration deterioration, activity limitation, and temporary or permanent damage to the appearance cause psychological distress (22, 23). However, the effect of distress disclosure level on the quality of life of patients with SLE has not been reported.

It has been reported that the social support of the rural elderly affected the health-related quality of life through the mediating effect of self-disclosure (24). Meanwhile, the self-disclosure of college students played a mediatory role in social support and subjective wellbeing (25). Brown et al. (26) also pointed out that social support affected HIV disclosure, and HIV-positive status disclosure concerns affected the quality of life (20). In addition, a study by Logie et al. (27) has proved that HIV disclosure mediated the effect of social support on the quality of life through a structural equation model. Therefore, we speculated that social support may affect the quality of life indirectly through disclosure.

Patients with SLE have been found to have greater psychological distress; it has been revealed that anxiety seriously affected the quality of life of patients with SLE (28), Conceio et al. (29) also found that psychological treatment can alleviate patients’ anxiety and depression and thus improve their quality of life. But there is also a study showing no relationship between anxiety and quality of life (30). The levels of disclosure and anxiety reflect psychological distress to a certain extent, which may jointly affect or moderate the quality of life (31). However, very limited studies have paid attention to the relationship between distress disclosure and anxiety.

To clarify the association of social support, distress disclosure, anxiety, and quality of life in female patients with SLE, we further explore the following contents: (1) occupation, SLEDAI, BMI, residence, marital status, disease duration, payment, education, monthly income, and age had significant differences in the quality of life of patients with SLE; (2) social support affected the quality of life; (3) distress disclosure, as a mediator variable, played a mediator role in the path from social support to quality of life; and (4) anxiety, as a moderator variable, played a moderator role in the effect of distress disclosure on quality of life.

## Materials and methods

### Design and participants

From 13/04/2021 to 24/05/2021, a cross-sectional convenient sampling survey was conducted in three Grade 3A hospitals in Hefei, Anhui province, China. A total of 237 female patients with SLE were recruited. Inclusion criteria: (1) the diagnosis meets the SLE classification criteria revised by the American College of Rheumatology (ACR); (2) voluntarily participates in the research with informed consent; (3) clear consciousness, certain comprehension, and language skills; (4) female; and (5) age ≥ 18 years old. Exclusion criteria: (1) severe infection or lupus encephalopathy; and (2) with other malignant tumors or serious life-threatening diseases.

The study participants were mainly 30–60 years old (42.14 and 13.86), with a short duration of disease (≤5 years) and a moderate income ($299–$598). A total of 44.3% of patients were with moderate and severe disease activity of SLE.

### Procedure

The inpatients from the Department of Rheumatology and Immunology at the First Affiliated Hospital of Anhui Medical University and Anhui Provincial Hospital were investigated after providing informed consent. R-CG and LW, two trained graduate student majoring in nursing, conducted this study independently and obtained informed consent from department leaders, teachers, and patients before the test. Within the department, we used a convenient sampling method and chose clean, relatively interference-free rooms as survey sites, avoided treatment, dine, and lunch times. The questionnaire was filled in anonymously. The subjects were required to answer the questionnaire truthfully and independently, and the questionnaire was withdrawn on the spot. If the patients could not complete the questionnaire independently, the researchers assisted them to complete it. It will take 5–10 min to complete the questionnaire based on the current situation. The integrity of the questionnaire was checked on the spot and corrected in time. A total of 249 questionnaires were received, the recovery rate of the questionnaire was higher than 95%, and 237 female patients with SLE were finally included.

### Measures

Demographic characteristics: A total of 11 demographic variables were included, including age, body mass index (BMI), duration, SLE disease activity index (SLEDAI), residence, payment (medical insurance), education, marital status, monthly income, occupation, and duration of drugs.

### Variables and instruments

Lupus Quality of Life (LupusQoL): Lupus QOL is a measure developed by McElhone et al. (32) in 2007 to assess the quality of life of patients with SLE in Great Britain, and it has been also translated into 77 languages for use in 51 countries (33). LupusQoL was used to evaluate the disease-specific quality of life. Its sensitivity was relatively high and increased SLE-specific items and measurement properties are better (34) as compared to the SF36 scale. The difference between the outcomes by the SF-36 and disease-specific health-related quality of life was previously reported (35), although there was comparability between the traditional SF-36 scale and the LupusQoL scale (36). A study has shown that LupusQoL was more suitable for measuring the quality of life of patients with SLE (37). A 5-point scale (score range of 0–4) was applied to assess the disease-related conditions in the last 4 weeks. It contains eight domains with a total of 34 items. Specifically, physical health, planning, pain, intimate relationships, burden to others, emotional health, body image, and fatigue. Each dimension can be converted to 0–100 (original score/number of entries/4) × 100 (38). The total score of the quality of life was expressed by the sum of the scores of each dimension (after conversion), or the total score was calculated directly by using the above method (39). This study used the original total score for analysis, the total original score range was 0–136. Lu et al. (34) of China formed the Chinese version of LupusQOL after cultural adjustment, with good internal consistency (0.811–0.965), good reliability, and validity. Cronbach’s α of the complete scale was 0.919 in this study.

Social Support Rate Scale (SSRS): SSRS was used to assess the level of social support, it contains 10 items with three dimensions: objective support, subjective support, and utilization of support. The higher the score, the higher the perceived social support. It has high reliability and validity. SSRS has shown good psychometric properties, with Cronbach’s coefficients ranging from 0.89 to 0.94 in each dimension (40). In this study, Cronbach’s α was 0.692.

The Distress Disclosure Index (DDI): The DDI was described by Kahn and Hessling (41), they found that the average score of female college students was 42.21 and the standard deviation was 9.16. This study applied the revised version of DDI by Li et al. Cronbach’s α coefficient is 0.866. It is used to evaluate the degree of self-disclosure of patients. The scale consists of 12 items, each item is 1–5 points, and the total score range from 12 to 60. The higher the score, the higher the degree of disclosure, suggesting negative emotional handling with good psychometric properties (42). Cronbach’s α coefficient range from 0.89 to 0.95 (43), and it was 0.965 in this study, indicating that the internal consistency of the scale was excellent.

Self-Rating Anxiety Scale (SAS): SAS is a self-reporting tool for assessing anxiety levels. It consists of 20 entries, each with a score of 1–4. SAS has demonstrated good psychometric properties (44). Wang et al. (45) tested localization and validity; Cronbach’s α coefficient was 0.931. A total score of 40 is the critical point, the higher the score, the more serious the anxiety. Cronbach’s α was 0.715.

### Ethic approval and consent to participate

The oral informed consent of all patients was approved by the Ethics Research Committee of the Second Affiliated Hospital of Anhui Medical University (reference number: YX2021-088).

### Statistical analysis

EpiData 3.1 software was applied for data input. SPSS 23.0 was used for data analysis. Quantitative variables were represented by the mean and standard deviation (SD), and categorical variables were expressed as counts and percentages. A Pearson correlation analysis was carried out among variables, including LupusQoL, age, duration, SSRS, DDI, and SAS. We conducted a prior hierarchical regression analysis to examine the influencing factors of SLE patients’ quality of life, taking into account that demographic variables may interfere with the model. Age, BMI, disease duration, residence, marital status, monthly income, payment, education, occupation, and SLEDAI were incorporated into the model as control variables. To test the moderated mediation effect among SSRS, DDI/SAS, and LupusQoL, we performed a multiple regression analysis. To further clarify whether SAS played a moderating role, it was verified by PROCESS model 5, model 7, and model 14, respectively. When model 14 was used in SPSS macro, the moderated mediator effect model was verified. Simple slope analysis showed the interaction effect among SAS and DDI on quality of life, the path coefficient of moderated mediation model was given. The number of bootstrap samples for percentile bootstrap confidence intervals was 5,000. The statistical results were tested by a two-tailed test (*p* < 0.05).

## Results

### Demographic characteristics and main variables of all female patients with systemic lupus erythematosus

The average DDI of female patients with SLE was lower than the normal for college students, 73 patients (30.8%) were higher than the normal for female college students. Compared with college students, DDI levels in patients with SLE were as follows: low level: 100 (42.2%), medium level: 78 (32.9%), and high level: 59 (24.9%).

The scores (mean and standard deviation) of lupus quality of life in eight dimensions were as follows: physical health: 75.4 and 16.94; pain: 75.46 and 18.97; planning: 68.53 and 17.22; intimate relationships: 84.39 and 18.84; burden to others: 49.68 and 23.87; emotional health: 75.04 and 16.23; body image: 73.76 and 21.24; and fatigue: 56.17 and 17.02. The score of intimate relationships was the highest, and burden to others was the lowest. The original total score of lupus quality of life was 95.86 and 16.05. Table 1 shows that LupusQoL had statistical significance in occupation.

**TABLE 1:** Demographic characteristics of LupusQoL (*N* = 237).

Variables	n (%)	LupusQoL		*t*/F	*p*
		Mean	SD		
		95.86	16.05		
Lupus nephritis				0.40	0.69
No	167 (70.5%)	96.13	16.49		
Yes	70 (29.5)	95.24	15.07		
Residence				–1.57	0.12
Rural	124 (52.3%)	94.33	18.01		
Town	113 (47.7%)	97.55	13.47		
Payment (medical insurance)				1.98	0.14
Self-supporting	39 (16.5%)	99.05	13.54		
Residents	157 (66.2%)	94.39	16.60		
Staff	41 (17.3%)	98.46	15.69		
Education				1.92	0.13
Primary or below	85 (35.9%)	93.54	14		
Middle school	97 (40.9%)	95.65	18.49		
Technical degree	34 (14.3%)	98.59	15.08		
College or above	21 (8.9%)	101.86	11.32		
Marital status				1.20	0.31
Unmarried	37 (15.6%)	96.89	18.99		
Married	194 (81.9%)	95.88	15.54		
Divorced	3 (1.3%)	99.00	3.		
Widowed	3 (1.3%)	79.00	11.79		
Monthly income ($)				1.68	0.17
≤299	68 (28.7%)	93	18.43		
299–598	113 (47.7%)	95.81	15.42		
598–897	51 (21.5%)	99.57	12.68		
≥897	5 (2.1%)	98.20	22.91		
Occupation				3.99	0.01*
On-the-job	74 (31.2%)	99	16.68		
Students	15 (6.3%)	92.60	21.09		
Farmer/ self-employed	52 (21.9%)	99.44	11.96		
Unemployed/ retired	96 (40.5%)	92.02	15.90		
Duration of disease				0.70	0.62
≤2	72 (30.4%)	96.90	16.29		
3–5	41 (17.3%)	99.17	14.80		
6–10	68 (28.7%)	94.10	15.43		
11–15	25 (10.5%)	94.76	13.48		
16–20	12 (5.1%)	94.67	15.26		
>20	19 (8.0%)	93.32	22.89		

*p < 0.05.

SD, Standard deviation; BMI, Body Mass Index; SLEDAI, Systemic Lupus Erythematosus Disease Activity Index.

### Correlation analysis of the distress disclosure index, self-rating anxiety scale, social support rate scale, and lupus quality of life in female patients with systemic lupus erythematosus

As shown in Table 2, LupusQoL had a strong correlation with age, SLEDAI, SSRS, DDI, and SAS. The quality of life was positively correlated with social support, while other variables were negatively associated with social support. There was no significant correlation between SAS and SSRS, but it was significantly related to DDI and quality of life. The correlation between the quality of life and the duration of the disease was not statistically significant.

**TABLE 2:** Correlation analysis among variables in female patients with SLE.

Variables	Mean	SD	LupusQoL	Age	Duration	SLEDAI	SSRS	DDI	SAS
LupusQoL	95.86	16.05	1						
Age	42.14	13.86	–0.22**	1					
Duration	8.16	7.37	–0.10	0.29**	1				
SLEDAI	9.25	4.95	–0.22**	0.05	0.12	1			
SSRS	40.33	5.85	0.26**	–0.10	–0.12	–0.02	1		
DDI	34.30	13.18	–0.29**	0.27**	0.09	–0.01	–0.46**	1	
SAS	34.53	5.68	–0.46**	0.21**	0.02	0.13*	–0.10	0.18**	1

*p < 0.05; **p < 0.01.

LupusQoL, Lupus Quality of Life; SLEDAI, Systemic Lupus Erythematosus Disease Activity Index; SSRS, Social Support Rate Scale; DDI, The Distress Disclosure Index; SAS, Self-Rating Anxiety Scale.

### The hierarchical regression analysis on lupus quality of life

Demographic variables affect the SLE quality of life (*F* = 3.18, *p* = 0); age, SLEDAI, and payment affect SLE patients’ quality of life and have a disturbing effect on the model. After controlling age, SLEDAI, and payment, SSRS has a positive effect on the quality of life (β = 0.61, *p* = 0). DDI, SAS, and DDI*SAS had a negative impact on the quality of life (β = –3.31, *p* = 0.02; β = –1.12, *p* = 0; β = –0.01, *p* = 0.02), as shown in Table 3.

**TABLE 3:** Hierarchical regression analysis on LupusQoL.

Variables	Model 1	Model 2	Model 3	Model 4	Model 5
(constant)	103.09	81.56	90.40	125.94	123.62
BMI	0.41	0.32	0.306	0.36	0.34
Age	–0.24*	–0.20*	–0.17	–0.08	–0.09
Duration of disease	–0.06	–0.02	–0.04	–0.10	–0.09
Residence	3.14	2.58	2.56	1.91	2.24
Education	–0.11	0.36	0.45	0.61	0.30
Marital status	1.40	0.76	1.22	0.88	0.92
Monthly income	0.02	–0.58	–0.43	–0.31	–0.36
Payment	–1.66	–2.13	–2.96	–3.48*	–3.40
Occupation	–1.21	–1.16	–1.08	–0.84	–0.75
SLEDAI	–0.67***	–0.67***	–0.68***	–0.51**	–0.51**
SSRS		0.61***	0.44*	0.39*	0.30
DDI			–3.31*	–2.65*	3.71
SAS				–1.12***	–0.65*
DDI*SAS					–0.01*
R^2^	0.12	0.17	0.19	0.33	0.35
ΔR^2^	0.12	0.05	0.02	0.14	0.02
F	3.18	4.19	4.38	8.54	8.51

*p < 0.05; **p < 0.01; ***p < 0.001.

SLEDAI, Systemic Lupus Erythematosus Disease Activity Index; SSRS, Social Support Rate Scale; DDI, The Distress Disclosure Index; SAS, Self-Rating Anxiety Scale.

### The test of moderated mediation effect

Age and disease activity index were used as control variables. Regression analysis detected the moderated mediating effect model. SSRS had a positive predictive effect on the lupus quality of life, while SLEDAI and DDI had the opposite effect. In addition, SAS had a negative predictive effect on the quality of life. The DDI and SLEQOL had a significant negative correlation (β = –0.14, *p* = 0.05). Although the coefficient of social support and quality of life decreased after adding DDI, it remained positively significant (β = 0.14, *p* = 0.05), indicating that DDI plays a partial mediating role between social support and quality of life. Quality of life was influenced by the interaction between DDI and SAS (β = –0.19, *p* < 0.01). SAS acts as a moderator, moderates the effect of DDI on quality of life, as shown in Table 4.

**TABLE 4:** The multiple regression analysis on LupusQoL.

Variables	LupusQoL			DDI			LupusQoL		
	B (95%CI)	β	t	B (95%CI)	β	t	B (95%CI)	β	t
Age	–0.21 (–0.35, –0.07)	–0.18	2.98**	0.21 (0.11, 0.32)	0.22	3.92**	–0.10 (–0.023, 0.03)	–0.09	1.54
SLEDAI	–0.66 (–1.05, –0.27)	–0.20	3.36**	–0.08 (-0.38, 0.22)	–0.03	0.54	–0.44 (–0.79, –0.09)	–0.14	2.50*
SSRS	0.65 (0.32, 0.98)	0.24	3.91**	–0.99 (–1.24, –0.74)	–0.44	7.75**	0.38 (0.05, 0.70)	0.14	2.26*
DDI							–0.17 (–0.32, –0.02)	–0.14	2.19*
SAS							–1.05 (–1.36, –0.74)	–0.37	6.63**
DDI*SAS							–0.04 (–0.07, –0.02)	–0.19	3.45**
F	13.22***			27.55***			19.75***		
R^2^	0.13			0.25			0.32		
ΔR^2^	0.15			0.26			0.34		

*p < 0.05; **p < 0.01; ***p < 0.001.

LupusQoL, Lupus Quality of Life; SLEDAI, Systemic Lupus Erythematosus Disease Activity Index; SSRS, Social Support Rate Scale; DDI, The Distress Disclosure Index; SAS, Self-Rating Anxiety Scale.

**FIGURE 1:**
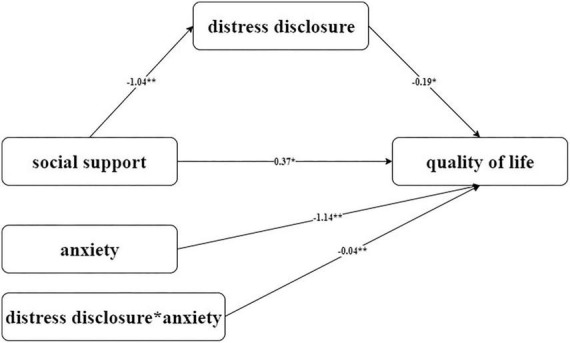
Hypothetical model of moderated mediator mediated by DDI and moderated by SAS. *p < 0.05; **p < 0.01. LupusQoL, Lupus Quality of Life; DDI, The Distress Disclosure Index; SSRS, Social Support Rate Scale; SAS, Self-Rating Anxiety Scale.

**FIGURE 2:**
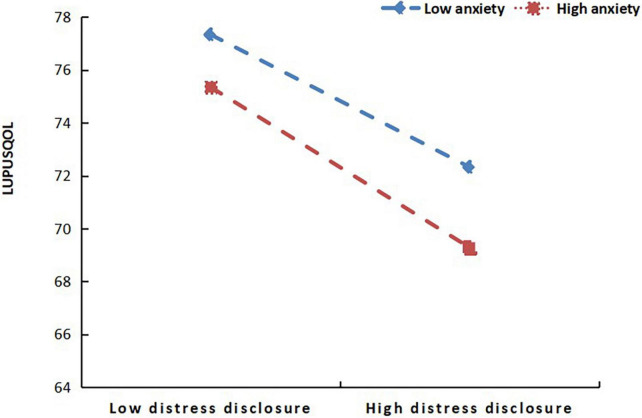
Moderating effect of self-rating anxiety scale (SAS) on the pathway from the distress disclosure index (DDI) to lupus quality of life (LupusQoL).

To further clarify the moderation effect of SAS in this mediation model, we applied Model 14 (a moderated mediator) in PROCESS macro to do the Bootstrap test. Figure 1 shows the model regression coefficients. In the mediator effect model of SSRS-DDI-LupusQoL, DDI was selected as the mediator variable; social support directly affected lupus quality of life. The direct effect was significant, DDI played a partial mediator role in the mediation model. The total effect was 0.57, the mediation effect was 0.19, and the mediation effect ratio was 33.3%. In the mediating effect model, when SAS was used as a moderating variable of the DDI-LupusQoL pathway, SAS had a moderating effect. Furthermore, a simple slope test found that compared with individuals with high anxiety level, individuals with low anxiety level had a more significant positive predictive effect of self-disclosure on the quality of life, indicating that with the increase in anxiety level, the positive predictive effect of self-disclosure on the quality of life gradually decreased, as shown in Figure 2.

Therefore, social support affected the lupus quality of life; some of them were directly affected, followed by distress disclosure as a mediator, and finally moderated the effects of distress disclosure on the quality of life through anxiety.

## Discussion

The quality of life of patients with SLE could not be well-controlled even in remission (46), however, the quality of life should be improved pertinently. Occupation, payment age, and disease activity may influence the quality of life of patients with SLE. The disease burden of lupus patients can affect their quality of life, but working or having a certain income can help (3, 47). Elera et al. also contend that sociodemographic factors, such as older age, poverty, and a low education level, are the primary predictors of poor quality of life in patients with SLE (48). According to this study, quality of life was negatively correlated with disease activity, which was consistent with previous research; the higher the disease activity, the worse the quality of life (38, 49). Furthermore, a previous study found that the quality of life was positively correlated with the duration of the disease; however, this was not found in this study. Therefore, the influence of disease duration on lupus quality of life needs to be demonstrated further (50).

This study indicated that social support, distress disclosure, and anxiety, were predictors of the lupus quality of life. After eliminating the interference of the age and disease activity index, social support has a positive influence on quality of life; and distress disclosure, and anxiety have a negative influence on the quality of life. Social support may be one of the direct factors affecting the quality of life of patients with lupus. Self-disclosure partially mediated the relationship between social support and quality of life. Anxiety moderated the latter half of the impact of social support on quality of life.

The social support of patients with SLE is correlated with their quality of life, which may be because higher social support means patients get more subjective and objective help and utilization of support, which increases patients’ confidence in facing difficulties, improves treatment compliance and is conducive to the improvement of their quality of life, which is consistent with the results of previous studies (51). In this study, self-disclosure was negatively correlated with quality of life, which may be due to the fact that patients with SLE suffer great pain in many aspects, including physiology, disease, treatment methods, and psychosocial function, which has a great impact on the quality of life, patients with higher levels of self-disclosure may suffer more pain and have a greater impact on the quality of life and are more willing to disclose. Currently, there is still no cure for SLE, which is prone to depression, anxiety, and other emotions of varying degrees during the disease (52); negative emotions are not conducive to the remission of disease and may reduce the quality of life. Although research has confirmed the impact of age on quality of life (53), a study by Khanna et al. (54) showed that the quality of life of patients with SLE was not affected by age and duration of disease. In this study, the third step of the multiple regression analysis found that age did not affect the quality of life, which was different from the results of the first test; so the effect of age on the quality of life of female patients with SLE needs to be further studied. Also, literature displayed that the quality of life was positively correlated with the duration of the disease (48), but it was not found in this study, which is consistent with Khanna et al.’s (54) findings.

At present, scholars generally agree on the role of social support in promoting the quality of life (55); social support was the only positive predictor in this study. The level of DDI can reflect patients’ willingness to disclose, investigating the level of DDI was conducive to a more comprehensive evaluation of psychological status. This study pointed out the significance of DDI in female patients with SLE. On the one hand, SSRS directly affected the quality of life; on the other hand, SSRS indirectly affected the lupus quality of life through the mediator role of DDI. DDI played a negative role in predicting the quality of life in female patients with SLE. Currently, the scope of application of DDI in various diseases was limited, there are few studies on the distress disclosure and the moderator effects of DDI in patients with SLE, which provided many possibilities for the study of it.

In the moderated mediating effect model, SAS was significantly correlated with DDI and quality of life, but not with SSRS, these results suggested that SAS may be involved in the effect of DDI on quality of life, but excluded the connection with SSRS, it confirmed the moderated mediator model. In addition, SAS played a moderating role in the pathway of DDI on quality of life, the moderator effect as a whole was a negative predictor of quality of life. Anxiety was also common in the study of various disease, it was a significant predictor of the quality of life (56). A study (57) suggested that social anxiety played a moderator role in the expectation of being liked and self-disclosure. The study of SAS as a moderator variable is still at an initial stage.

There was no clear conclusion about the internal relationship among SSRS, SAS, DDI, and quality of life. A study by Rotheram-Borus et al. (58) has shown the relationship among social support, quality of life, disclosure, and depression through the structural equation model, there was also a link among social support, physical and mental health, disclosure, and psychological resilience (27). A survey (59) showed the correlation between self-disclosure and social support, anxiety, and quality of life, but the relevant contents, especially the four variables, were still immature. This study focused on the relationship between the psychosocial status and quality of life of female patients with SLE, it can effectively supplement this relevant part and provide important reference value. Of course, male patients can be included in the future to demonstrate and elaborate on this direction in the longer term.

## Limitations

First, the survey methods adopted in this study are all in the form of self-reports, thus there may be subjective assumptions that have an impact on the results. There are many forms of self-disclosure and differences in expression, and patients were unable to pour out their emotions in a short period of time to test the predictive value of disclosure to mental health. A high level of disclosure may be an important event stimulation, or it may be a positive coping style, which is closer to expressing desire or demand and cannot fully reflect the mental health status of patients. Second, this study cannot determine causality due to its cross-sectional design. Third, this study did not make a detailed study of the eight dimensions of the quality of life, and only used the original score to study; its rigor remains to be confirmed, and there was a lot of room for research on its internal relationship, which can be discussed in depth in the future. To improve the quality of life in patients with SLE, interventions should focus on psychosocial factors such as social support, self-disclosure, and anxiety.

## Conclusion

The key finding of this study is that distress disclosure has a partial mediator effect between social support and quality of life in female patients with SLE, while anxiety has a moderator effect between distress disclosure and quality of life. When distress disclosure is increased, the positive impact of social support on quality of life may be diminished. At low anxiety level, the slope was relatively small, and the quality of life of lupus was higher with the decrease in DDI score. With the increase in anxiety level, the positive predictive effect of self-disclosure on quality of life decreased gradually. In future studies, our goal is to improve the quality of life of patients with SLE by improving the level of social support. In addition, we will focus on the mediating role of distress disclosure and the moderating role of anxiety. This could be an important development in the field.

In a theoretical sense, our study explores a new understanding of the impact of social support on the quality of life in patients with SLE. Furthermore, using distress disclosure as the mediating variable, we discovered that social support decreased distress disclosure in patients with SLE, and that distress disclosure ultimately had a negative impact on quality of life, whereas anxiety had a moderating effect on self-disclosure and quality of life.

Clinical and practical implications for practice, this study describes the relationships between the four variables proposed in this study, which may aid researchers in better understanding the mechanisms that influence the quality of life. Positive psychological and lifestyle interventions for patients with SLE and their families can therefore mitigate the negative effects of poor quality of life in patients with SLE. This theory will also have implications for policy in terms of social support and anxiety. Effective measures for improving the quality of life of patients with SLE include raising their awareness of the need for assistance, increasing social support, and avoiding excessive disclosure of distress. Medical institutions should also take a multidisciplinary approach to provide comprehensive care, including psychological care, to patients with SLE who are anxious. This intervention can help patients rebuild their perception of the disease, allowing them to actively receive treatment, control disease activities on time, and avoid a decline in the quality of life. Nursing plans should include SLE self-care health education.

## Data availability statement

The raw data supporting the conclusions of this article will be made available by the authors, without undue reservation.

## Ethics statement

The studies involving human participants were reviewed and approved by the oral informed consent of all patients was approved by the Ethics Research Committee of the Second Affiliated Hospital of Anhui Medical University (reference number: YX2021-088). The patients/participants provided their written informed consent to participate in this study.

## Author contributions

R-CG and G-CW designed the study. R-CG, LW, P-LS, NS, and MH gathered the data. R-CG and LW run and described the statistical analyses. R-CG, LW, and G-CW interpreted the data and wrote the manuscript. All authors approved the final version of the manuscript and edited the manuscript.
